# Spontaneous shape transition of Mn*_x_*Ge_1−_*_x_* islands to long nanowires

**DOI:** 10.3762/bjnano.12.30

**Published:** 2021-04-28

**Authors:** S Javad Rezvani, Luc Favre, Gabriele Giuli, Yiming Wubulikasimu, Isabelle Berbezier, Augusto Marcelli, Luca Boarino, Nicola Pinto

**Affiliations:** 1 INFN - Laboratori Nazionali di Frascati, Via Enrico Fermi 54, Frascati, Italy; 2 Advanced Materials Metrology and Life Science Division, INRiM (Istituto Nazionale di Ricerca Metrologica), Strada delle Cacce 91, Torino, Italy; 3 IM2NP, CNRS, Aix-Marseille Université, Marseille (AMU), France; 4 School of Science and Technology, Geology division, University of Camerino, Camerino, Italy; 5 CNR - Istituto Struttura della Materia and Elettra-Sincrotrone Trieste, Basovizza Area Science Park, 34149 Trieste, Italy; 6 RICMASS - Rome International Center for Materials Science – Superstripes, Via dei Sabelli 119A, 00185 Roma, Italy; 7 School of Science and Technology, Physics division, University of Camerino, Camerino, Italy

**Keywords:** nanowires, semi-metallic Ge–Mn alloy, strain-induced growth

## Abstract

We report experimental evidence for a spontaneous shape transition, from regular islands to elongated nanowires, upon high-temperature annealing of a thin Mn wetting layer evaporated on Ge(111). We demonstrate that 4.5 monolayers is the critical thickness of the Mn layer, governing the shape transition to wires. A small change around this value modulates the geometry of the nanostructures. The Mn–Ge alloy nanowires are single-crystalline structures with homogeneous composition and uniform width along their length. The shape evolution towards nanowires occurs for islands with a mean size of ≃170 nm. The wires, up to ≃1.1 μm long, asymptotically tend to ≃80 nm of width. We found that tuning the annealing process allows one to extend the wire length up to ≃1.5 μm with a minor rise of the lateral size to ≃100 nm. The elongation process of the nanostructures is in agreement with a strain-driven shape transition mechanism proposed in the literature for other heteroepitaxial systems. Our study gives experimental evidence for the spontaneous formation of spatially uniform and compositionally homogeneous Mn-rich GeMn nanowires on Ge(111). The reliable and simple synthesis approach allows one to exploit the room-temperature ferromagnetic properties of the Mn–Ge alloy to design and fabricate novel nanodevices.

## Introduction

Metallic and semimetallic nanowires (NWs) have attracted vast interest in nanoscale electronic and spintronic systems due to their thermal [1], electrical [2], and magnetic [3] properties. Low-dimensional materials have unique electronic properties that can be tuned via geometrical or structural modifications [4–8]. Also, the tunability of the spin degrees of freedom in semiconducting materials offers a great potential for future spintronic applications. However, to achieve a reliable injection and detection of spin-polarized electrons in spintronic devices, appropriate heterostructures between semiconductors and magnetic alloys [9–10] need to be formed. Hence, a tailored growth process that preserves the injection efficiency and high Curie temperature is necessary.

Mn–Ge alloys epitaxially grown on Ge substrates have been shown to be promising candidates for such spintronic systems [11–13]. Transition metal germanides that have sharp interfaces and a tunable Schottky barrier, in particular, can advantageously replace silicides as an indispensable part of microelectronics [14–15]. In particular, the manganese germanide phase Mn_5_Ge_3_ is a semimetallic compound that has attracted attention due to its giant magnetoresistance and large spin polarization, which make it a proper candidate for spintronics applications [16–17]. The growth of Mn*_x_*Ge_1−_*_x_* alloys on Ge wafers, and in particular of Mn_5_Ge_3_, has been studied extensively in recent years [18–24]. The high Curie temperature of Mn_5_Ge_3_ (≈296 K), in particular, can make it suitable to exploit ferromagnetic properties in everyday applications [25–26].

Several studies have been published on manganese germanide systems, from deposited films to free-standing nanoclusters and Mn embedded in a Ge matrix, but only few works have been devoted to one-dimensional Mn–Ge systems [11–13,20–23,27]. Semiconducting and alloyed nanowires can be obtained via chemical methods [28–29] or via vapor–solid–liquid (VLS) and, less frequently, vapor–solid–solid (VSS) mechanisms. A metallic droplet (liquid or solid) acts as a catalyst, in chemical vapor deposition (CVD), or as a seed, in molecular beam epitaxy (MBE), for the NW growth [7,30–31]. By using these techniques, NWs are grown away from the substrate, usually in a tilted direction, and size distribution and geometry strongly depend on the growth dynamics [32–33]. Furthermore, it is known that the catalyst introduces uncontrolled and unwanted contamination inside the crystal lattice of the wires. For instance, Au, generally used as catalyst for the growth of various semiconductor NWs, acts as a deep-level trap in germanium bulk and NWs, modifying the electronic transport properties [5].

Strain-induced elongation is a mechanism [34] that can lead to either epitaxial or endotaxial formation of quantum wires [35]. In this method, wires are obtained via epitaxial growth of a strained wetting layer followed by annealing at high temperature. However, only few studies have been dedicated to strain-induced elongation mechanisms leading to the formation of semiconducting nanowires, such as Ge on Si substrates, or the endotaxial growth of transitional metal silicides (e.g., CoSi_2_) [34,36]. In these studies, the NWs exhibit a narrow diameter distribution, in contrast to those obtained by VLS, which commonly have wider range due to the droplet size distribution.

In the present work, we report a spontaneous morphology modification, from islands to nanowires, in Mn-rich GeMn nanoparticles. The growth is initiated via reaction of a thin Mn wetting layer, evaporated by MBE, with a Ge(111) substrate. Morphology and microstructure of the NWs have been studied by scanning electron microscopy (SEM), X-ray diffraction (XRD), and high-resolution transmission electron microscopy (HRTEM). We demonstrate that the thickness of the Mn layer and the annealing conditions finely control the shape transition, resulting in NWs up to ≃1.5 μm length with uniform width and homogeneous composition.

## Experimental

Samples were grown in a MBE chamber with a base pressure of 3 × 10^−11^ Torr. Ge(111) wafers were ultrasonically cleaned in methanol and trichloroethylene, followed by removal of the native oxide using sulfuric acid and formation of a volatile oxide by dipping in H_2_O_2_/NH_3_OH/H_2_O. Prior to Mn deposition, Ge wafers were annealed at 400 °C for 30 min to remove the overgrown oxide. Then, a Ge buffer layer of 80 nm was deposited at 350 °C, using a Knudsen cell, and let to cool down to 60 °C for Mn deposition. Two to nine monolayers of Mn were deposited at 60 °C. The films were annealed immediately after deposition (in the same chamber) at 650 °C for 15–30 min and then cooled down rapidly to room temperature (RT). Samples were studied using SEM, XRD, and HRTEM. XRD data were collected by means of a PW 1830 diffractometer in Bragg–Brentano geometry. A long fine-focus Cu tube was operated at 40 kV and 25 mA with a graphite monochromator. Step-scan diffractograms were collected in the 2θ range of 3–70° with 0.02° step and 3 s/step counting time. For HRTEM analysis, focused ion beam (FIB) lamellae were prepared using a dual-beam FIB. The lamellae were oriented along the elongation direction. The lamellas were ultimately thinned during a last step using a FIB based on an inert-gas source working at low energy to prevent sample amorphization. HRTEM investigations were performed at the CP2M microanalysis center (Marseille, France) on a JEOL JEM 2010 F URP22 instrument using a 200 keV primary energy electron beam.

## Results and Discussion

**Figure 1:**
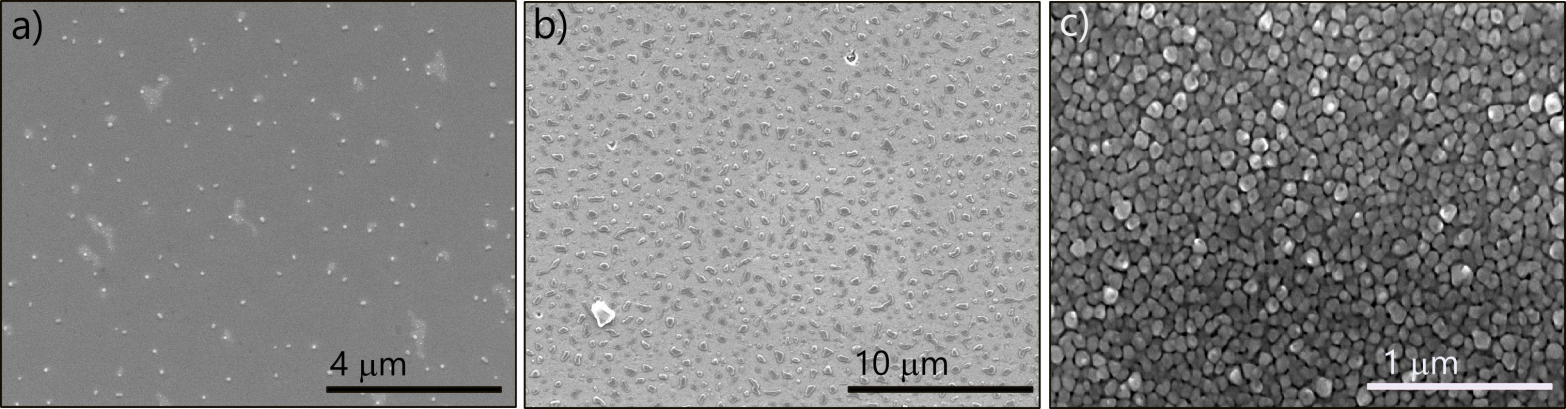
SEM images of the morphological evolution of strained Mn wetting layers with different thickness after annealing at 650 °C for 15 min: (a) 2.2 ML, (b) 6.7 ML, and (c) 9 ML.

High-temperature annealing of the evaporated Mn thin films on Ge(111) results in a significant change of the film morphology with the appearance of nanostructures onto the surface. The features of the structures are related to the Mn layer thickness and the duration of the thermal annealing. Annealing of 2.2 ML of thickness of Mn at 650 °C for 15 min produces both round islands, with a mean diameter of 139 nm (Figure 1a), and widespread milky and irregularly shaped regions. These regions contain few large islands on the border area surrounded by small islands. This distribution of islands suggests an incomplete ripening process of the small islands. The ripening cannot take place completely due to the low density of small islands as a consequence of the reduced thickness of the Mn layer. A sparse but uniform distribution of bigger islands and milky regions has been found everywhere on the sample surface.

A thicker Mn layer (6.7 ML), under the same annealing conditions, yields larger islands with a mean lateral size of ≈400 nm and a homogeneous distribution on the surface area (Figure 1b). Their irregular shape is the result of a coalescence process of smaller nanoparticles (NPs). Further increase of the Mn layer thickness to 9 ML results in a closely packed film of agglomerated islands with a relatively uniform size distribution (≃100 nm) completely filling the surface (see Figure 1c).

**Figure 2:**
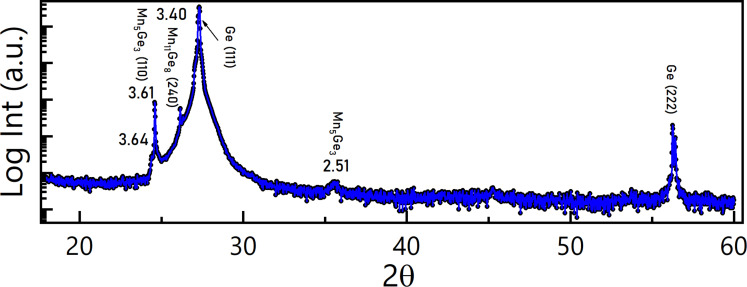
X-ray diffraction pattern of the 9 ML thick Mn wetting layer, upon annealing at 650 °C for 15 min. Both Mn_5_Ge_3_ and Mn_11_Ge_8_ phases have been detected, the former being dominant.

XRD in Bragg–Brentano geometry has been carried out on these islands on the annealed 9 ML thick Mn film. The detected signal combines information from the nanoparticles present on the surface and the underlying Ge(111) substrate. The XRD pattern exhibits two main peaks related to the (111) and (222) crystallographic planes of Ge (Figure 2). The spectrum shows two less intense peaks corresponding to *d* values of 3.61 and 3.40 Å which are related to the (110) plane of Mn_5_Ge_3_ and the (240) plane of Mn_11_Ge_8_, respectively. Finally, a small component corresponding to a *d* value of 2.51 Å assigned to Mn_5_Ge_3_ with the *c* axis out of plane of the substrate could be observed. Despite representing much less than 1% of the intensity of the Ge(111) peaks, they can be clearly discerned. Although the NPs are small, the full width at half maximum (FWHM) of the diffraction peaks is as low as 0.05°, which is indicative of a good crystallinity of the grown Mn*_x_*Ge_1−_*_x_* NPs. The absence of the other characteristic peaks of Mn_5_Ge_3_ and Mn_11_Ge_8_ can be due to the perfect iso-orientation of all the NPs present on the film surface (Figure 1c). Similar results have been observed on thinner samples, even if the detected signal has been less intense due to the reduced thickness of the Mn layer.

**Figure 3:**
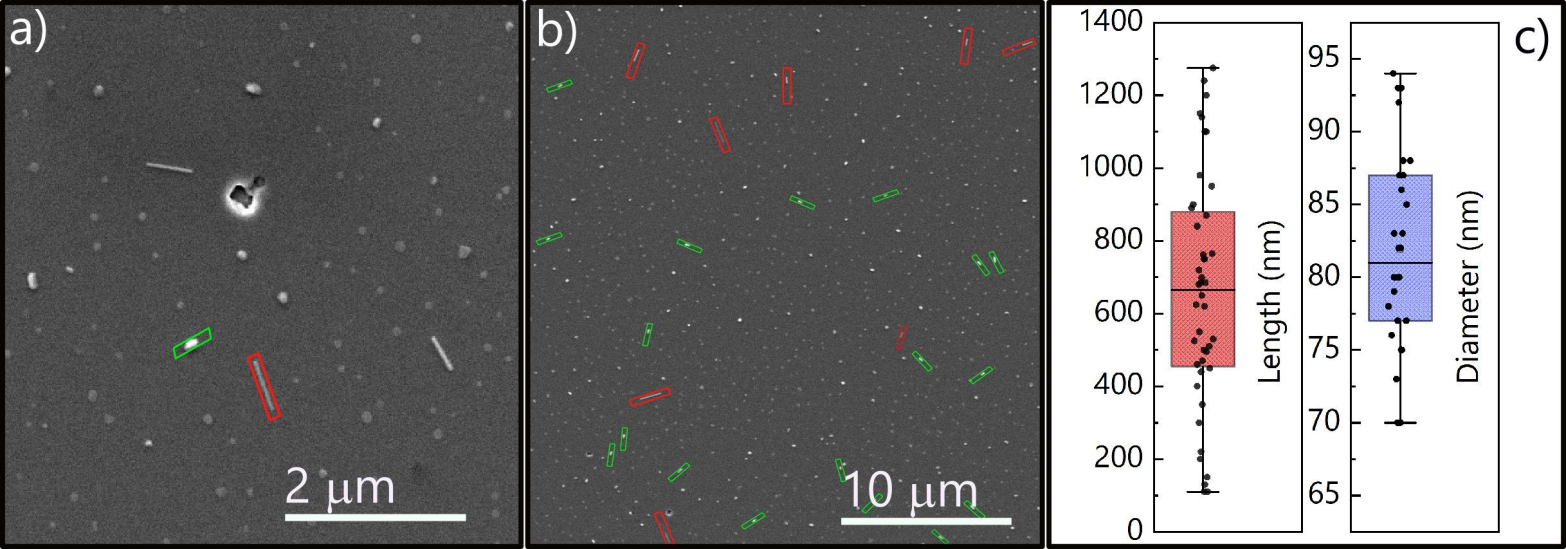
SEM images of the surface after deposition of 4.5 ML Mn on Ge(111) wafer and annealing at 650 °C for 15 min at (a) higher and (b) lower magnification. The two populations of 3D islands can be clearly distinguished with several small round islands and some long and short NWs. c) Length and diameter distribution of the NWs. The whiskers define minimum and maximum value of each dimension, while the boxes show the 25th and 75th percentile of the distribution with the middle line representing the average value of ≈700 nm for the length and ≈80 nm for the diameter.

The results of SEM and XRD investigations are in agreement with previous studies that have proved the formation of Mn-rich GeMn phases (e.g., islands) during the co-epitaxial growth of Mn and Ge by MBE on Ge substrates [19–24,27,37]. Even at low substrate temperatures and low Mn contents in the Ge*_x_*Mn_1−_*_x_* alloy (*x* ≤ 5%), the formation of Mn_11_Ge_8_ and Mn_5_Ge_3_ phases is energetically favoured with a preference for the latter [19–24,27,37]. Under the same annealing conditions, for a Mn film thickness of 4.5 ML a drastic change of the resulting morphology is observed with the spontaneous formation of elongated nanostructures, dispersed among smaller islands similar to those observed in the 2.2 ML sample (Figure 1a). Their length spreads from few hundred nanometers to ≃1.1 μm, with a mean value of ≃700 nm (Figure 3a,b). Hereafter, we will refer to these objects as nanowires. Despite the relatively wide range of length, these NWs exhibit a narrow distribution in the lateral size, with a mean value of (80 ± 10) nm (Figure 3c).

**Figure 4:**
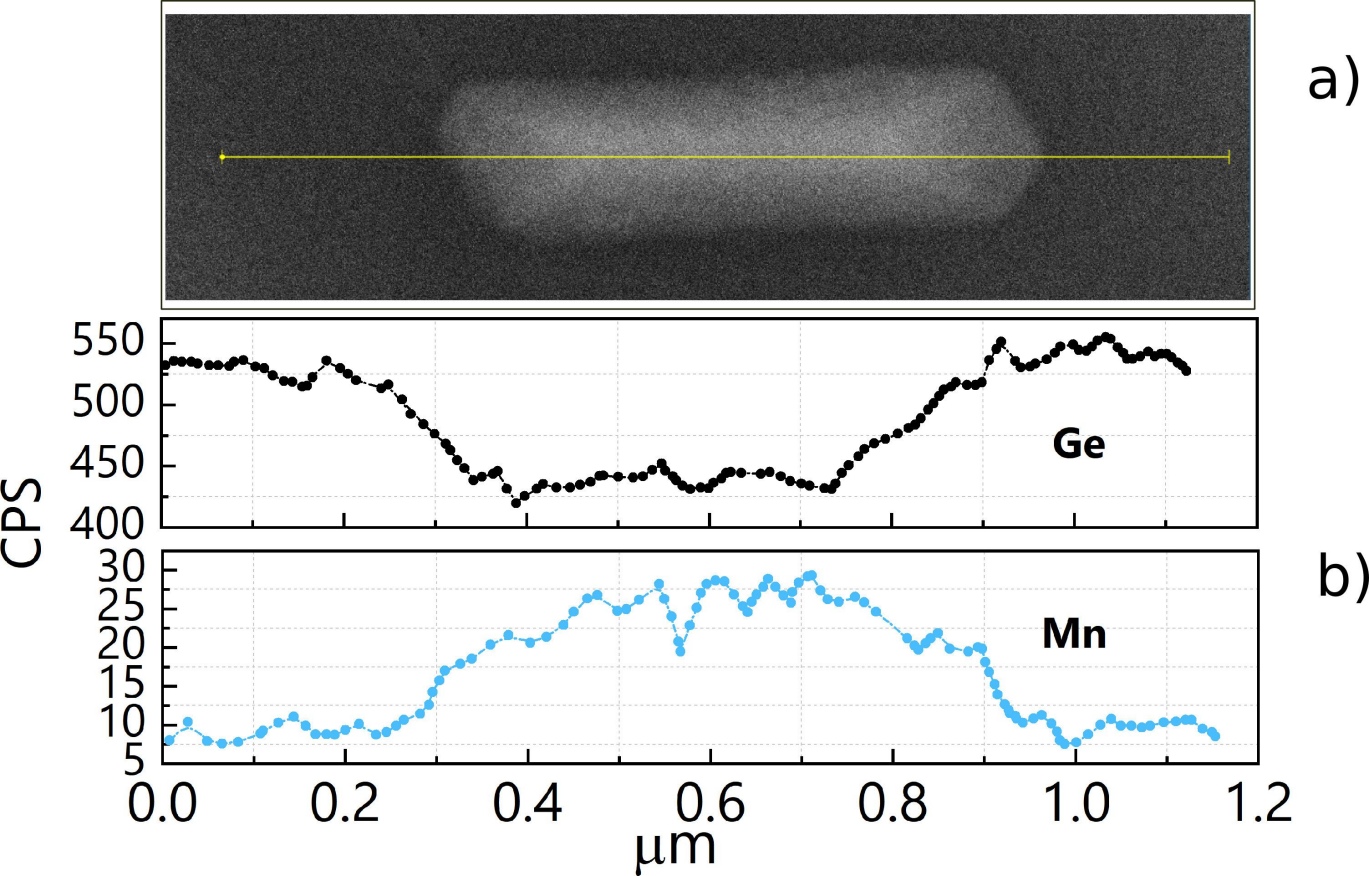
(a) SEM image of a typical NW, ≃700 nm long, obtained by deposition of a 4.5 ML thick Mn film followed by annealing at 650 °C for 15 min. (b) Mn and Ge EDX line profiles along the yellow line drawn in (a).

The EDX analysis of a NW in plane-view configuration (Figure 4a) exhibits both Mn and Ge with a homogeneous distribution along the NW length (Figure 4b). When approaching the extremities of the NW, the concentration of Mn tends to decrease linearly with the distance before attaining a constant value, with a specular behaviour for the Ge content (Figure 4b).

**Figure 5:**
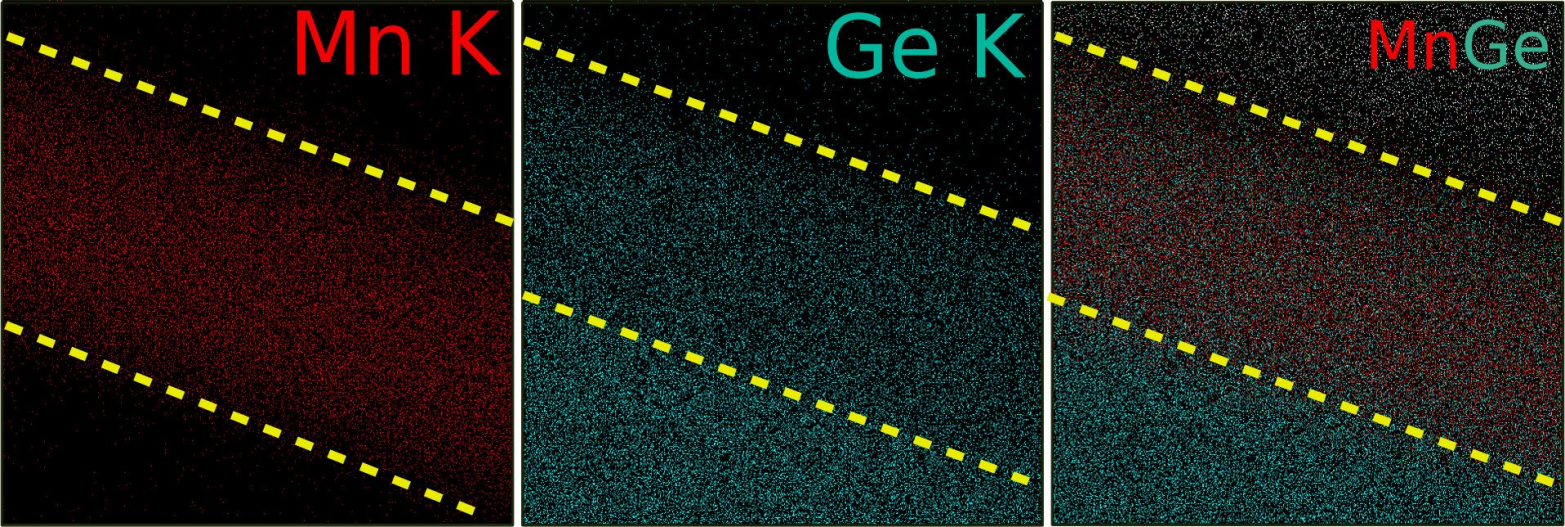
EDX elemental map (Kα line), carried out on a cross-section of a NW on the surface of the 4.5 ML thick Mn film showing the distribution of Mn, Ge, and Ge + Mn in the NW and in the buffer layer. In all panels the dashed lines highlight the NW region.

This linear decrease is in agreement with the faceted shape of the NWs. The facet formation at the extremities of the nanowires is in agreement with previously observed results in the case of silicides [2]. Here, it is explained by the progressive reduction of the NW thickness, which consequently contributes less and less the closer you get to the edges (while the contribution from Ge substrate increases proportionally). We conclude that the NW composition is homogeneous throughout the total volume of the NW from the elemental map recorded on a cross-section of a NW of the same sample showing uniform distribution of Ge and Mn in the whole NW volume (Figure 5). Here, the Mn signal appears stronger than that of Ge, confirming the formation of a Mn-rich Ge–Mn alloy.

**Figure 6:**
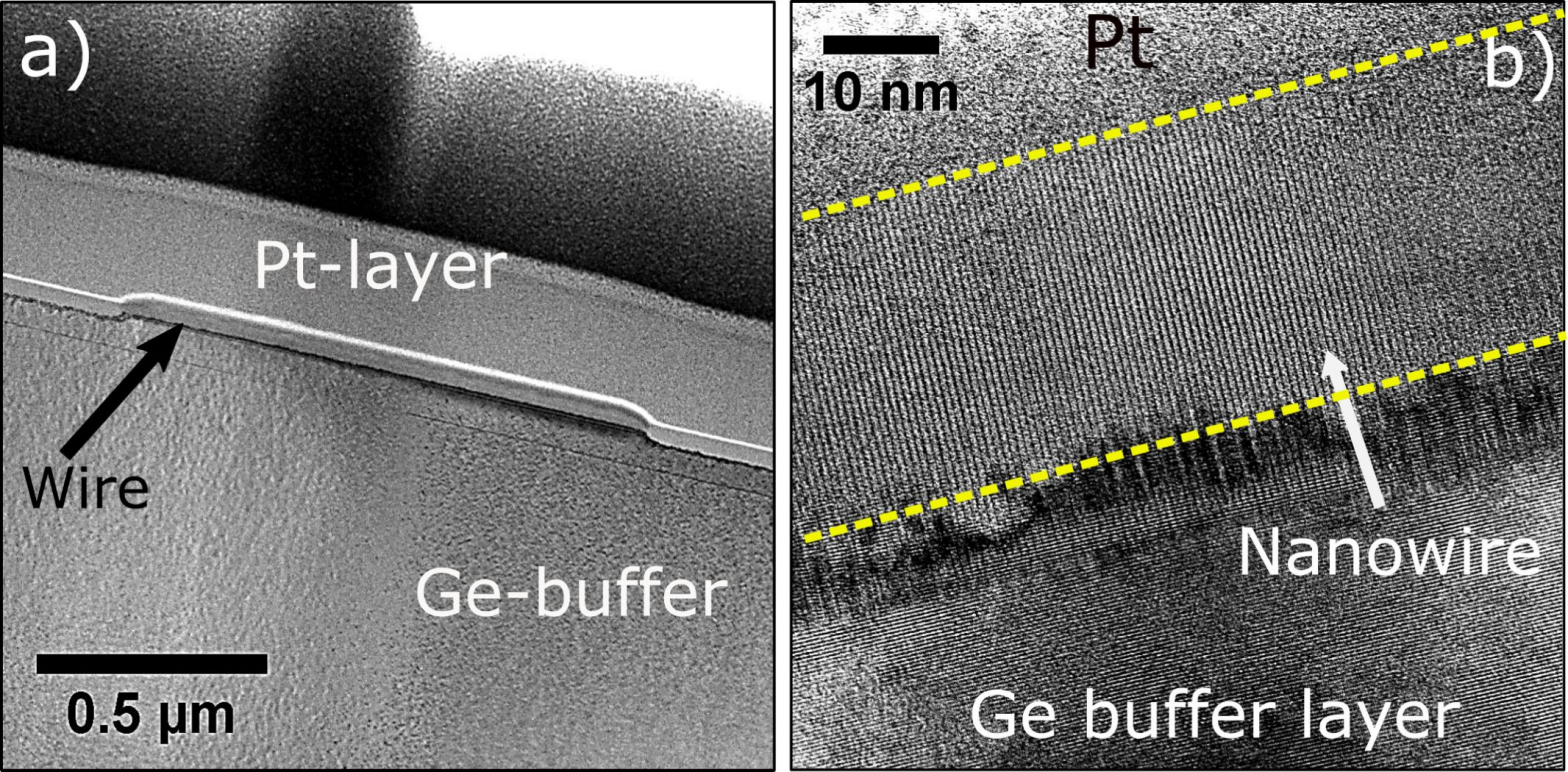
Cross-section image of the NW along its length. (a) TEM image of a 1.15 μm long NW capped with a Pt layer. (b) HRTEM image along [110] zone axis. The dashed lines highlight the height of the NW of ≈36 nm.

These results raise two important issues: First, the NW growth is not driven by a VLS-type mechanism since, under our conditions, the Mn concentration is constant throughout the volume of the NWs. In contrast, a growth driven via Mn seeds would have induced a Mn gradient with a higher concentration close to the Mn droplet, which in our case was not detected [7,30–31]. Second, the width of the NWs is remarkably constant with a very narrow size distribution. In addition, a cross-section HRTEM image along the [110] zone axis (Figure 6) reveals three different areas, that is, the Ge substrate where the Ge(100) planes can be seen, the interfacial layer with variable thickness (around 5 nm), where intermixing between Ge and Mn takes place, and the monocrystalline GeMn top layer. Identification of the GeMn phase in the top layer (NW) by SAED or by inferring from the interplanar distance was not possible because of the complexity of the Ge/Mn phase diagram. These results show that the NW is monocrystalline and epitaxially grown on the substrate [38]. The question is to understand how these Mn-rich Ge–Mn NWs are formed under our experimental conditions (i.e., high-temperature annealing of Mn wetting layers).

While several works have been published so far on the seeded VLS growth of Mn-rich Ge–Mn NWs [7,39–40] or on the eutectoid growth of Mn-rich Mn*_x_*Ge_1−_*_x_* nanocolumns with strongly inhomogeneous Mn distribution [25], to best of our knowledge, this is the first report on the spontaneous growth of Mn*_x_*Ge_1−_*_x_* NWs that do not result from seeded growth and/or phase transformation. Considering all the features of the NWs observed here, we suggest that they are formed by a two-step process. First, the growth of 3D islands to relieve the epitaxial strain between the 2D Mn layer and the substrate occurs (significantly increasing the critical thickness for crystallographic defects nucleation). Second, there is the spontaneous elongation of 3D islands also induced by strain relaxation, which is the driving force leading to the final growth of the NWs, considering that strained heteroepitaxial layers are inherently unstable [33]. During the heteroepitaxial growth of GeMn alloys, Mn-rich precipitates have been detected in Mn*_x_*Ge_1−_*_x_* DMS films on moderately heated Ge wafers [19,21,23,25,27,37,41]. However, islands of Mn-rich Ge–Mn phases could easily nucleate on the surface upon high-temperature annealing of the Mn wetting layer. At low Mn thicknesses, small islands are expected to be strained and their nucleation onto the film surface occurs during the early stage of the annealing process, driven by strain between the epilayer and the substrate as detected in several heteroepitaxial systems, such as Ge on Si [42–44], InAs on GaAs [45], Co silicide [36], and silicides with different metals [35]. Such a mechanism is expected to occur in our Mn layers deposited on Ge(111) substrates, due to the large lattice mismatch between Mn and Ge.

Moreover, it has been predicted and experimentally confirmed that these small strained islands grow linearly in width and length, up to a critical size related to the maximum deformation and shape energy that the islands can build up. The rapid increase of this energy cannot be sustained by the islands keeping a regular symmetric shape [34,36]. In fact, passing a critical point, the islands grow asymmetrically. The width will gradually reduce, approaching an asymptotic value, while the length will further increase. As a consequence, the aspect ratio will rise. Within this framework, the effect of the Mn wetting layer can also be explained as the reservoir of adatoms required to reach and pass the critical point while not influencing the mechanism of island formation. Based on the simplistic model proposed by Tersoff and Trump [34], shape and dimensions of the islands are controlled by the interplay between contributions from the relevant surface and interface energies, *E*_s_, and the energy variations due to the elastic relaxation, *E*_r_ [34]. The sum of these two energy terms, *E* = *E*_s_ + *E*_r_, represents the total increment of the island energy. In this scenario, the transition from larger islands to elongated wires occurs due to the elastic relaxation of the strained islands. Assuming, for simplicity, a rectangular island [34] for which *s*, *t*, and *h* are the width, length, and height, respectively, the minimization of the island energy requires the fulfilment of the condition *s* = *t* = α_0_, with:

[1] $$\alpha_{0}=e\phi h\exp\left(\Gamma /ch\right),$$

where *e* is the Neper number, ϕ = *e^−3/2^*cot(θ), with θ the contact angle of the island facet with the substrate. Γ = γ_e_csc(θ) − γ_s_cot(θ) with γ_e_ and γ_s_ are the surface energy per unit area of the edge facet and the substrate, respectively. 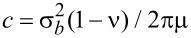, where σ*_b_*, ν and μ are the stress tensor in the plane, the Poisson ratio, and the shear modulus of the substrate, respectively [34]. The value of Γ/*ch* controls the final distribution and size of the islands. In particular, if Γ/*ch* ≫ 1, then α_0_ becomes too large to reduce the edge-to-area ratio. While the mechanism described here applies to the heteroepitaxial growth, it may be considered valid also for solid-phase epitaxy, which occurs in our system (Mn wetting layer on Ge), since the key role in the process is the mechanism of diffusion of adatoms (i.e., Mn) occurring also during the annealing process. According to this model, the optimal island shape that minimizes the island energy is a squared one (i.e., *s* = *t*) until the condition *s* = *t < e*α_0_ is fulfilled. On the contrary, for dimensions *s* = *t* ≥ *e*α_0_ the squared shape is unstable and a transition to a rectangular shape will result. As the island grows, the aspect ratio (i.e., *t*/*s*) becomes larger, finally resulting in the formation of a NW. The elongation in one direction, while achieving the optimal dimension in the perpendicular direction, enables islands to release half of their relaxation energy [34].

**Figure 7:**
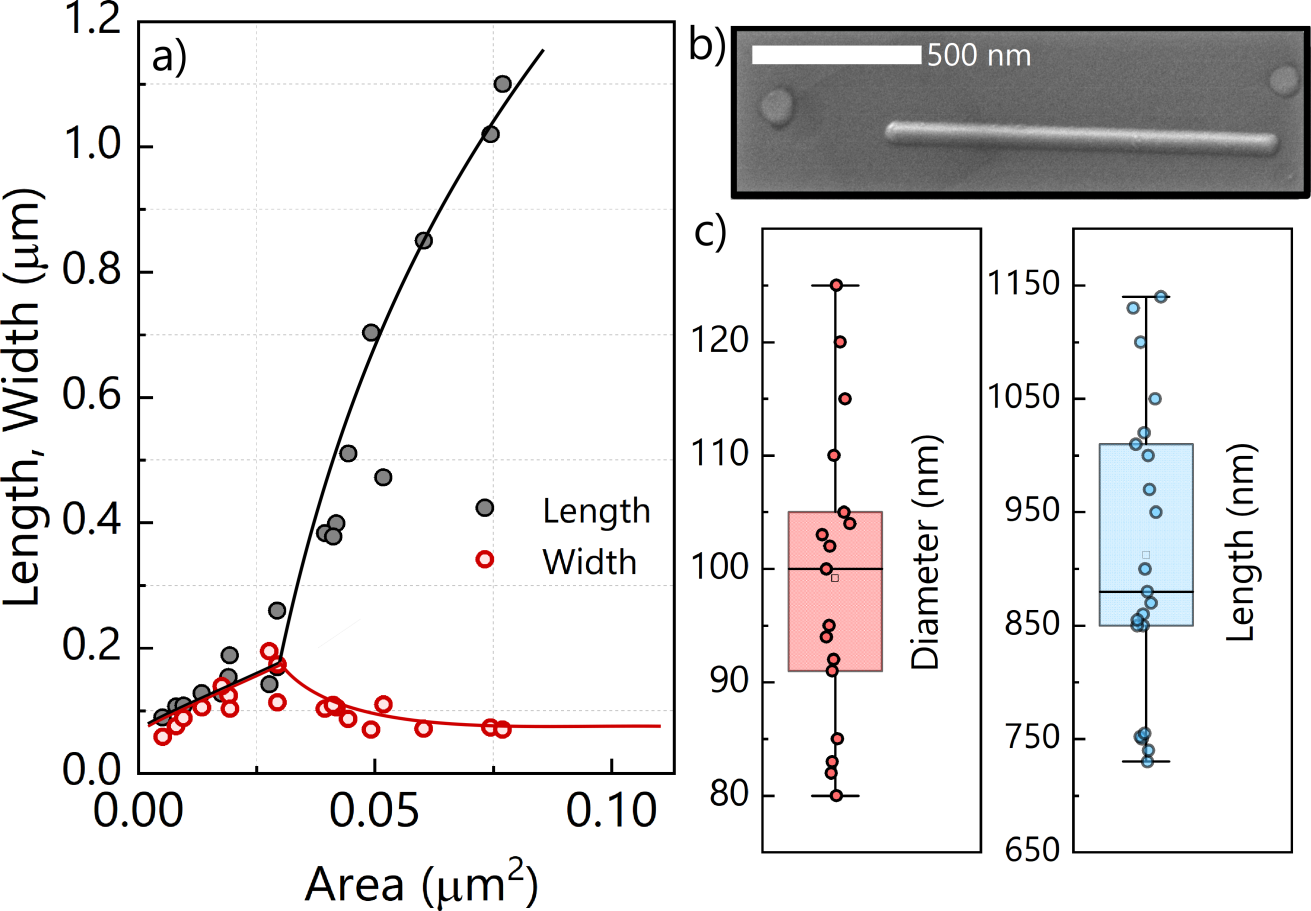
(a) Length (*t*) and width (*s*) of Mn_5_Ge_3_ islands, formed on the sample surface of the 4.5 ML thick Mn layer upon annealing at 650 °C for 15 min, as functions of the island area. Deviation from a regular island shape (diverging branches) occurs at *s* = *t* ≈ 170 nm. At area values of ≈0.075 μm^2^, the width approaches the optimal value of *s* ≃ 80 nm (red line) while the length continues to grow (black line). Lines are guides to the eyes. (b) SEM image of the typical morphology of a NW (after annealing for 30 min at 650 °C) to facilitate the comparison with experiment. (c) Diameter and length distribution of NWs (conditions similar to that in (b)). The whiskers show the minimum and maximum value of NW dimensions, while the colored boxes show the 25th and 75th percentile of the distribution with the middle line representing the average value.

In order to verify the compatibility of the model to our system, we have measured the *s* and *t* values of the islands observed for the 4.5 ML thick Mn film (see Figure 3a,b) as a function of the area. We have found that islands grow linearly up to the critical size of *s* = *t* ≈ 170 nm, corresponding to a value of α_0_ ≃ 60 nm (Figure 7a). Once the islands exceed this critical dimension, the islands start to elongate rapidly and their width approaches the optimal value of *s* ≃ 80 nm (Figure 7a). We have calculated an island aspect ratio as large as *t*/*s* ≃ 17 in our sample. These results confirm the compatibility of the nanowire growth with the strain-induced elongation mechanism, also observed in CoSi_2_ and endotaxially grown Ge NWs [7,32,36]. However, the specific parameters of the Mn/Ge system yield other sizes and aspect ratios. We explain the higher aspect ratio and critical size by the relatively high annealing temperature used in our experiments. Furthermore, possible modulations of dimensions can be attributed to the initial strain (i.e., lattice mismatch) between the wetting and buffer layers.

For a longer annealing time (30 min) at the same temperature and thickness, we observe a shift of *s* and *t* to higher values while the surface density significantly reduces. On average, NWs are ≈900 nm long and ≃100 nm wide (see Figure 7c), while the length can reach ≈1.5 μm. This behaviour corresponds to a ripening process as expected under these experimental conditions. In the absence of a continuous supply of Mn adatoms, the number of the adatoms available for the formation of the elongated islands is limited, which restricts the ripening process as well. However, the variation of the NW size distribution can also be attributed to the modulation of the surface energies and to the value of the parameter Γ, which can modify the aspect ratio of the wires and the critical value of α_0_. A detailed analysis of the influence of growth and annealing temperatures and annealing time on size and aspect ratio of the NWs is in progress to quantify the formation mechanisms and the driving forces at work in our experiments. However, the effect of both temperature and lattice matrix properties (especially the latter) requires further detailed analysis and experiments.

## Conclusion

We have demonstrated that a thin Mn wetting layer, evaporated at low temperature on Ge(111), can undergo a spontaneous shape transition, from islands to nanowires, upon high-temperature annealing. We have shown that the Mn thickness is a crucial parameter for the initiation of the shape transition. Experimental results demonstrate that 4.5 ML is the crucial thickness of the Mn wetting layer. Our study has established that the wires are monocrystalline, uniform in width and compositionally homogeneous, and made of a single Mn-rich Mn–Ge alloy phase. Such NWs cannot be formed by a VLS-type growth. Based on our analysis, the best mechanism describing the solid-phase growth of our NWs is a strain-driven energetic mechanism, originally proposed in the literature for heteroepitaxial growth [34]. Our experimental outcomes qualitatively fit with the predictions of this theoretical model. The transition from islands to wires takes place at a critical island size of ≃170 nm. This critical dimension can be tuned by the experimental conditions (e.g., a prolonged annealing). Further systematic experiments are required to better quantify the morphological evolution as function of the experimental parameters. Nevertheless, to the best of our knowledge, the present study is the first experimental evidence for the spontaneous formation of monocrystalline Mn–Ge NWs on Ge(111), with constant lateral size and uniform composition, up to ≃1.5 μm in length. Considering that several Mn-rich Ge–Mn phases are usually ferromagnetic at room temperature, our results offer an alternative route to a simple and fast fabrication process of novel nanodevices, capable to exploit ferromagnetic properties.
